# Measuring Protein Aggregation and Stability Using High-Throughput Biophysical Approaches

**DOI:** 10.3389/fmolb.2022.890862

**Published:** 2022-05-16

**Authors:** Tristan O. C. Kwan, Stefan A. Kolek, Amy E. Danson, Rosana I. Reis, Ines S. Camacho, Patrick D. Shaw Stewart, Isabel Moraes

**Affiliations:** ^1^ National Physical Laboratory, Teddington, United Kingdom; ^2^ Douglas Instruments Ltd., Hungerford, United Kingdom

**Keywords:** protein aggregation, DLS, DSF, CD, crystallography, cryo-EM, drug discovery, high-throughput

## Abstract

Structure-function relationships of biological macromolecules, in particular proteins, provide crucial insights for fundamental biochemistry, medical research and early drug discovery. However, production of recombinant proteins, either for structure determination, functional studies, or to be used as biopharmaceutical products, is often hampered by their instability and propensity to aggregate in solution *in vitro*. Protein samples of poor quality are often associated with reduced reproducibility as well as high research and production expenses. Several biophysical methods are available for measuring protein aggregation and stability. Yet, discovering and developing means to improve protein behaviour and structure-function integrity remains a demanding task. Here, we discuss workflows that are made possible by adapting established biophysical methods to high-throughput screening approaches. Rapid identification and optimisation of conditions that promote protein stability and reduce aggregation will support researchers and industry to maximise sample quality, stability and reproducibility, thereby reducing research and development time and costs.

## Introduction

Human health is continuously challenged not only by external toxic substances but also by microorganisms such as pathogenic bacteria and viruses. Thus, understanding biology at a cellular and molecular level is paramount to human existence. Proteins are sophisticated molecular machines essential in all cellular processes. However, because of their complex intrinsic three-dimensional (3D) molecular network of hydrogen bonds, Van der Waals forces, hydrophobic interactions and disulfide links, proteins have a natural tendency to denature (unfold) or aggregate (formation of non-physiological dimers and higher-order aggregates). The pathways and mechanisms by which proteins unfold and aggregate are not yet fully understood. However, external factors such as temperature, pH, ionic strength, different buffer systems, mechanical stress or even the presence of “foreign bodies” like dust, glass particles and microscopic oil droplets can lead to structural and behavioural changes of the protein in solution.

Currently, there are many critical reasons why scientists need to monitor aggregation and stability of proteins in solution. Firstly, pure and stable protein material is always required for structural studies for any of the three most popular methods—X-ray crystallography, NMR spectroscopy, and cryo-electron microscopy (cryo-EM) (Chari et al., 2015; Kozak et al., 2016; Kotov et al., 2019; Kwan et al., 2019). Protein purification strategies and protocols are particularly dependent on the target protein that frequently misbehaves when isolated in solution. Therefore, the ability to quickly search chemical spaces that increase protein stability with minimal amounts of sample is critical to such biophysical applications. Over the last few decades, however, developments in gene editing tools, molecular biology, instrumentation, and light sources, along with many other multidisciplinary approaches, have enabled a *new age* in protein structure determination (Adli, 2018). Consequently, structural biologists have pursued more challenging targets such as integral membrane proteins and protein-protein complexes. Moreover, the structural biology community is also now strongly focused on time-resolved approaches to study protein dynamics and conformational changes over time (Kwan et al., 2020; Bonvin, 2021; Cohen, 2021). Secondly, critical to biopharmaceutical industry and biomedicine, protein unfolding and aggregation also poses many challenges in the development of biologics (therapeutic agents manufactured in living systems such as a bacteria, plant or animal cells) because of its impact on the final product quality in terms of safety, clinical efficacy, and immunogenicity (Ratanji et al., 2014; Roberts, 2014; Krause and Sahin, 2019). Presently, the most popular biologics in the market are monoclonal antibodies, cytokines, enzymes, and peptide hormones. Although these may offer many clinical advantages when compared with small molecule drugs, they are far more complex to develop and produce at a larger scale (Plant et al., 2014; Puetz and Wurm, 2019; Jovčevska and Muyldermans, 2020). By their intrinsic nature, protein-based drugs are extremely sensitive to physical and chemical environmental factors such as temperature, shear-forces, light, pH, glycosylation, and enzymatic action Rajan et al., 2021. Hence, probing protein aggregation during the development and manufacturing of protein-based drugs is essential to achieve a final product that is not only stable for long-term storage but also harbors fewer impurities that stimulate an anti-drug immune response (Ratanji et al., 2014; Svilenov et al., 2018; Ribeiro et al., 2019). Finally, production of high-quality protein is also important in many other industrial applications such as agriculture, food and beverage processing, production of cosmetics and biofuels, and in biotechnology applications such as biosensors and optogenetics (Zhang and Cohen, 2017).

Nevertheless, inherent to all these approaches is the protein sample quality in terms of purity and stability. Currently, a variety of biophysical techniques are available to study protein behaviour in solution. These range from simple approaches to complex methods that require both technical expertise and costly instruments. For example, Makowski et al. (2022) reviewed the use of a set of key bionanotechnologies for preparing functional and stable membrane proteins in diverse types of lipoparticles in combination with several biophysical assays, including affinity-capture self-interaction nanoparticle spectroscopy (AC-SINS). In a seminal study by Jain et al. (2017), 137 isotypematched IgG1 antibodies were evaluated with a dozen biophysical property assays to define the boundaries of drug-like behaviour for future studies. Screening multiple macromolecular constructs is distinct from the exploration of chemical space with a single target, and will not be considered further here.

In this perspective, we have summarised simple and readily available approaches protein aggregation and stability within a high-throughput (HTP) setting. These biophysical techniques are also capable of screening and probing the addition of specific ligands, additives or excipients to the protein of interest that can reduce its propensity to unfold or aggregate, as well as its susceptibility to proteolysis (Senisterra and Finerty, 2009).

## HTP Approaches for Measuring and Analysing Protein Stability

Automation and miniaturisation combined with multi-well microplates have enabled the development of HTP approaches where many samples (up to thousands) are tested in parallel under given conditions. Introduced by pharmaceutical companies in the early 1990s for the massive screening of potential “drug-like” compounds, HTP approaches are extensively used by scientists today in academic laboratories, research institutes and industry in a variety of applications (Macarron et al., 2011; Kwan et al., 2019; Kiriiri, et al., 2020).

**FIGURE 1:**
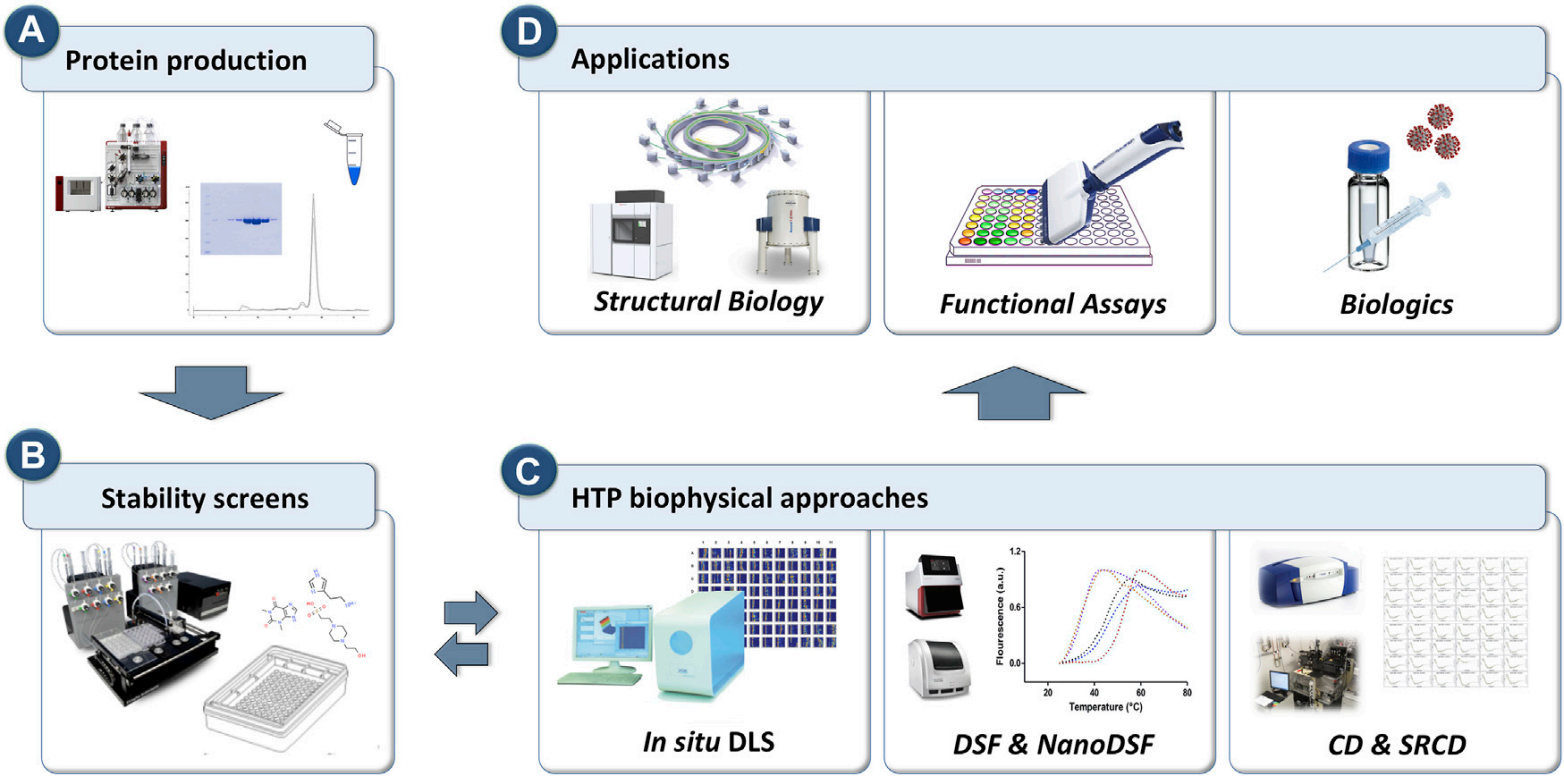
Schematic representation of the workflow for the HTP biophysical approaches to probe protein behavior for downstream and upstream applications. **(A)** Proteins are purified using standard purification strategies. **(B)** HTP screens are prepared to explore chemical space. **(C)** *in situ* Dynamic Light Scattering (DLS), differential scanning fluorimetry (DSF) and circular dichroism (CD) that can operate in HTP mode are used as primary screening tools to probe protein stability. **(D)** Three key applications where protein monodispersity and stability is crucial.

Recently, researchers have started to use HTP approaches to explore chemical space by testing protein samples against premixed sets of buffers, excipients, additives, salts, osmolytes, and co-factors (often referred as stability screens). Suitable stability screens are commercially available in 96- and 384-well format, or they can be assembled in-house. To maximise the throughput towards evaluation of protein behaviour in the presence of such stability screens or ligands, HTP approaches were also applied to simple but robust biophysical methods such as dynamic light scattering (DLS), differential scanning fluorimetry (DSF) and circular dichroism (CD). Each of these biophysical methods provides different types of information and, whether used as a standalone or integrated, they have proven to be noteworthy screening tools when probing protein aggregation or to identify conditions that promote protein integrity and stability in solution (Figure 1).

### HTP Dynamic Light Scattering

DLS is a common technique employed to assess the behaviour of proteins in solution. This simple yet powerful and non-invasive biophysical measurement correlates the random motion of particles in a fluid with their sizes. During a DLS experiment, particles in solution are illuminated by a visible and monochromatic laser light while a detector records the light scattered by the particles. The intensity of light fluctuates at a frequency that is determined by the particles’ size. Because proteins and other macromolecules in solution are not always spherical in shape and are always solvated, the size calculated by a DLS instrument is referred to as the “hydrodynamic radius”. The physical principles of light scattering and DLS have been extensively described in the literature (International Organization for Standardization, 1996; Lorber et al., 2012; Stetefeld et al., 2016; Kwan et al., 2019). For DLS experiments, the data are usually presented in terms of sample polydispersity. If the sample remains monodisperse and shows a peak at the expected size, the protein can be assumed to be stable in the chemical and physical conditions (temperature) used (Shiba et al., 2010). With the development of HTP approaches to DLS, the behaviour of proteins can now be determined in the presence of ligands, substrates, additives, and buffers in parallel at microlitre volumes in plate formats (Meyer et al., 2015; Kwan et al., 2019; Dauer et al., 2021) Moreover, the DLS measurement can be run over several time points, allowing degradation or changes in aggregation to be monitored over time. For example, we found that a bacterial ABC transporter, which is a membrane protein, tended to aggregate after purification. We used a 96-component screen to monitor aggregation over 9 h. The screen contained a set of conditions that has previously increased solubility and stability of proteins. We were able to identify 12 reagents that reduced aggregation of the ABC transporter (Supplementary Figure S1). Note that DLS provides extra information about the degree of aggregation, including the size of aggregates and the number of peaks, whereas other methods such as DSF give only a single numerical value corresponding to the “melting point” of the protein. HTP DLS provides a practical and simple probe for stability that can be implemented easily in the protein production workflow and downstream applications in structural biology such as crystallography and/or cryoEM. Finally, for the production of biologics, HTP DLS measurements can also be used to determine sample quality and be implemented as a quality control measurement between production batches (Hulse et al., 2013; Yu et al., 2013; Bhirde et al., 2018; Dauer et al., 2021).

### HTP Differential Scanning Fluorimetry

DSF, also commonly referred to as thermal-shift assay or ThermoFluor assay, is an inexpensive and highly sensitive biophysical technique that can be used to determine the thermal stability of a target protein in the presence or absence of ligands, substrates, and buffers. The DSF principle measures the protein thermal denaturation over time (typically about 1°C min^−1^) and is based on the relationship between protein stability and its Gibbs free energy of unfolding (ΔG_u_). When the amount of unfolded protein equals the amount of folded protein, the value of ΔG_u_ becomes zero and the system has reached what is known as the protein “melting point” temperature (T_m_). Proteins with higher stability have larger ΔG_u_ values and therefore a higher T_m_. The DSF experimental setup is very simple and typically does not require expensive equipment. The protein sample, in the presence of a highly sensitive fluorescent probe, also known as a “reporter” or extrinsic dye, is gradually heated over time (in order to trigger denaturation) from its stable baseline temperature. This eventually results in exposure of the hydrophobic regions that are usually buried within the protein’s folded structure. As more of the protein’s hydrophobic regions become exposed, an increasing number of dye molecules bind to the hydrophobic sites, resulting in an increase of the fluorescence signal. Once the protein reaches its maximum unfolded state, it starts to aggregate and precipitate, and consequently, the dye molecules dissociate and the fluorescence signal drops (Bai et al., 2019). In the majority of the instruments, the DSF output is displayed as a series of sigmoidal curves, also known as “melt curves”, where the point of inflection gives the T_m_ value (Supplementary Figure S2A). Although there are many fluorescent dyes available on the market, the most frequently used for soluble proteins is SYPRO Orange, which is inexpensive, highly sensitive and has low interference with small molecules. Its excitation/emission wavelength (480 nm/569 nm) is usually compatible with most filters available in fluorescent plate readers and RT-PCR machines. However, because SYPRO Orange also binds to the hydrophobic regions of lipids and detergent micelles, it is unsuitable for membrane protein studies. Instead, a reactive thiol-specific fluorochrome, N-[4-(7-diethylamino-4-methyl-3-coumarinyl)phenyl] maleimide (CPM), was introduced in 2008 (Alexadrov et al., 2008), which specifically reacts with the cysteine side chains that are usually buried in the interior of membrane proteins. Therefore, as the target membrane protein unfolds, the cysteines become exposed and the CPM fluorescence signal increases. Supplementary Figure S2B shows DSF results obtained for the membrane protein LacY with CPM dye in combination with five detergents that were identified from a detergent screen. Note, however, that the use of the CPM dye is associated with two limitations. One is its excitation/emission wavelength (387 nm/463 nm) that requires specific filters that usually are only available by request. The other limitation is that the protein of study must have cysteine residues in the transmembrane domains. More recently, a label-free DSF method known as nanoDSF was developed (Alexander et al., 2014; Gao et al., 2020; Real-Hohn et al., 2020). In this approach the protein’s intrinsic fluorescence, based on signals mainly from the native tryptophan residues, is measured at both 330 and 350 nm in low-volume capillaries, which produces more robust data in comparison to detection at a single wavelength. NanoDSF is especially useful for studying membrane proteins (Kwan et al., 2019; Gao et al., 2020) and viral particles (Real-Hohn, et al., 2020).

Either DSF or nanoDSF approaches are also used to optimise protein buffers for long-term storage, and to determine optimal conditions for protein refolding (Biter et al., 2016; Wang et al., 2017) and protein stability in solution, greatly aiding structural studies such as crystallisation and cryo-EM (Martinez et al., 2021). Finally, the DSF approach is also extensively used in early drug discovery platforms. Many proteins can be stabilised by the binding of low molecular weight ligands, usually resulting in an increase of the T_m_ and therefore, DSF is regarded as a simple and inexpensive, and popular method to assess protein-ligand interactions (Huynh and Partch, 2015).

The multi-well design of thermal cycling machines and fluorescent plate readers, including instruments that make use of small volume glass capillaries, allow DSF to be used as a HTP tool requiring relatively low (microliter range) sample volumes. A downside of DSF, however, is that larger macromolecular complexes may not unfold cooperatively (thus having multiple transition states), making stabilising conditions difficult to identify (Stark and Chari, 2016). This is particularly problematic if cryo-EM is the intended downstream process, as typically this is the method of choice for the structural elucidation of large protein complexes.

### HTP Circular Dichroism

CD is a biophysical technique that measures the differential absorption between left and right circularly polarised light by a chiral molecule. Polypeptide backbone information can be obtained from measurements taken in the far-UV region of 180–250 nm, due to the amide electronic transitions, and can provide estimates on the protein secondary structure content such as alpha-helices and beta-sheets (Kwan et al., 2019; Miles et al., 2021). The near-UV region of 260–300 nm is used to characterise the aromatic side chain transitions of tyrosine, tryptophan, and phenylalanine, which are often situated at binding sites, and thus measurements in this region provide valuable information regarding ligand interactions (Kwan et al., 2019).

The application of CD can be significantly broadened by the elevated photon flux of a synchrotron, which improves the signal-to-noise levels considerably, giving rise to a technique known as synchrotron radiation CD (SRCD). SRCD is capable of measuring at lower wavelengths, greatly improving the analysis of beta-sheet content and fold motifs, as well as at lower sample volumes and concentrations (Wallace et al., 2011; Hussain and Siligardi, 2016), which is a significant advantage when studying membrane proteins, for which high concentrations and volumes are difficult and costly to produce. Additionally, higher ionic solutions can be measured (up to 500 mM NaCl) due to the higher flux, which extends the buffer screening capability of conventional CD (Hussain and Siligardi, 2016). For example, beamline B23 at Diamond Light Source operates in a HTP manner (also referred to as HT-CD), with 96 or 384-well suprasil quartz plates available for data collection, in which protein samples of just 15 ul at 0.5 mg/ml can be measured allowing users to screen a wide range of buffers and/or ligands without the laborious task of changing and washing each cuvette (Hussain et al., 2016). Supplementary Figure S3 shows results obtained with HT-CD for two membrane proteins in combination with 12 detergents. CD data analysis is also swift due to the introduction of software that enables users to remotely process results quickly and easily (Hussain et al., 2015). Recent advances have led to the development of time-resolved SRCD (TR-SRCD), which is capable of subpicosecond temporal resolutions, providing insight into biological mechanisms at a molecular level (Auvray et al., 2019).

## Perspective Summary

Purified proteins are critical tools in a variety of research and industrial applications. However, these vital molecular machines are inherently unstable and prone to aggregation both *in vivo* and *in vitro*. Protein aggregates vary from small dimers to large assemblies that can be specifically formed during protein production and the storage process, including prolonged storage. Unfortunately, protein aggregates are usually irreversible and exceptionally stable. Therefore, effective strategies to quickly probe and quantify protein aggregation including protein stability *in vitro* are urgently needed. However, one of the major challenges is the absence of a single analytic approach that is able to cover the whole spectrum of protein behaviour *in vitro,* and therefore several different biophysical approaches are usually required.

Over the years, with the development of HTP technology, many traditional biophysical techniques such as DLS, DSF, and CD have evolved from standalone approaches, where samples used to be measured one at a time, to robust and sensitive methods able to analyse dozens of samples in parallel using minimal amounts of material and in standard workflows that can be applied to many targets. Each of these HTP biophysical methods has its own strengths and weaknesses (see Table 1), thus their applicability will depend on the information required.

**TABLE 1:** Summary of the strengths and limitations of the HTP biophysical methods presented in this perspective.

Method	Strengths	Limitations	Sample specification
Dynamic light scattering (DLS)	• Simple protocol and data analysis	• Measurements are affected by the introduction of air bubbles during sample preparation	Volume: 0.2 to 2 μl per well
	• Fast set up	• Measurements are highly sensitive to temperature and solvent viscosity (this note is for instruments without temperature control)	Concentration: 0.25 to 50 mg/ml
	• High throughput		
	• Low protein consumption	• Sometimes difficult to resolve polydisperse samples of similar sizes, e.g., monomers/dimers within 1–2 nm	Sample delivery: Multi-well plates
	• Low consumable costs		
	• Temperature range between 10 to 40°C		
	• Measurements can be taken over long time periods		
	• Direct measurement of aggregation		
	• Direct measurement of oligomerisation		
Differential scanning fluorimetry (DSF) *Fluorescence Dye-based (DSF)* and *Label Free DSF (NanoDSF*)	• Simple protocol and data analysis	• Difficult to interpret for larger macromolecular complexes	Volume: 50 μl per well or 10 μl per capillary
	• Fast experiment	• Fluorescence dye-based DSF	Concentration: 0.01 to 200 mg/ml (depending on the instrument used)
	• High throughput	-Requires the use of a fluorescent dye	Sample delivery: Multi-well plate or 10 μl glass capillaries
	• Low protein consumption	-Ligands and detergents may interact with the dye	
	• Low consumable costs	-The use of reducing agents interfere with certain dyes (e.g., CPM and SyproOrange)	
	• Inexpensive sample preparation	-Plate readers need to have the correct filter	
	• Rapid assessment of buffers, ligands and mutations in protein stability	• NanoDSF (label free DSF)	
		-Its signal is highly dependent of the protein aromatic residues such as tryptophan	
Circular dichroism (CD) and synchrotron radiation circular dichroism (SRCD)	• Reasonably easy to set up	• Requires high sample concentration if protein buffer has high salt concentration	Volume and concentration: 1 mg/ml in a volume of ∼25 μl when using a 0.1 mm cuvette
	• Relatively low amounts of sample		
	• Fast set up	• Limited to buffers and ligands that do not strongly absorb in the far-UV region	Sample delivery: Quartz single cuvettes or 96/384 multi-well plates made of fused quartz
	• Semi-high throughput		
	• Low consumable costs	• Not applicable to cloudy or colloid samples	
	• Temperature melt range between 5 to 95°C		
	• Accurate protein secondary structure quantification		
	• Direct measurement of protein conformational changes/dynamics		

One of the major advances in drug discovery and development has been its synergy with a wide variety of such biophysical methods (Raynal et al., 2014; Renaud et al., 2016; Holdgate et al., 2019; Kiriiri, et al., 2020). Today, modern medicine relies heavily on the use of conventional drugs (synthetic small chemical substances) and biologics (e.g., proteins, antibodies and small peptides) as therapeutic agents. While genomic and proteomic approaches have uncovered an increasing number of potential druggable proteins, compelling Pharma businesses to adopt target-based drug discovery strategies, many challenges still remain. These include poor understanding of the mode of action of the target protein and its interaction with the therapeutic agent, as well as difficulties associated with the manufacturing of high-quality protein-based biologics in terms of purity and stability (Davis 2020; Emmerich et al., 2021). In this perspective, we sought to direct the reader’s attention to the application of conventional and simple biophysical methods within automated HTP settings that are able not only to assess protein stability and integrity in solution but also to probe protein behaviour in the presence of multi-parametric screens of buffers, excipients, additives, salts, osmolytes, and co-factors. Moreover, these HTP biophysical approaches can make significant contributions to downstream applications such as X-ray crystallography, NMR and cryo-EM, as well as to the upstream applications in drug discovery, development and formulation.

## Data Availability

The original contributions presented in the study are included in the article/Supplementary Material, further inquiries can be directed to the corresponding authors.
