# Supplementary figures and images for: Measuring Protein Aggregation and Stability Using High-Throughput Biophysical Approaches
Source: Front Mol Biosci. 2022 May 16;9:890862. doi: 10.3389/fmolb.2022.890862 (PMC9149252; doi:10.3389/fmolb.2022.890862)

**(A)**

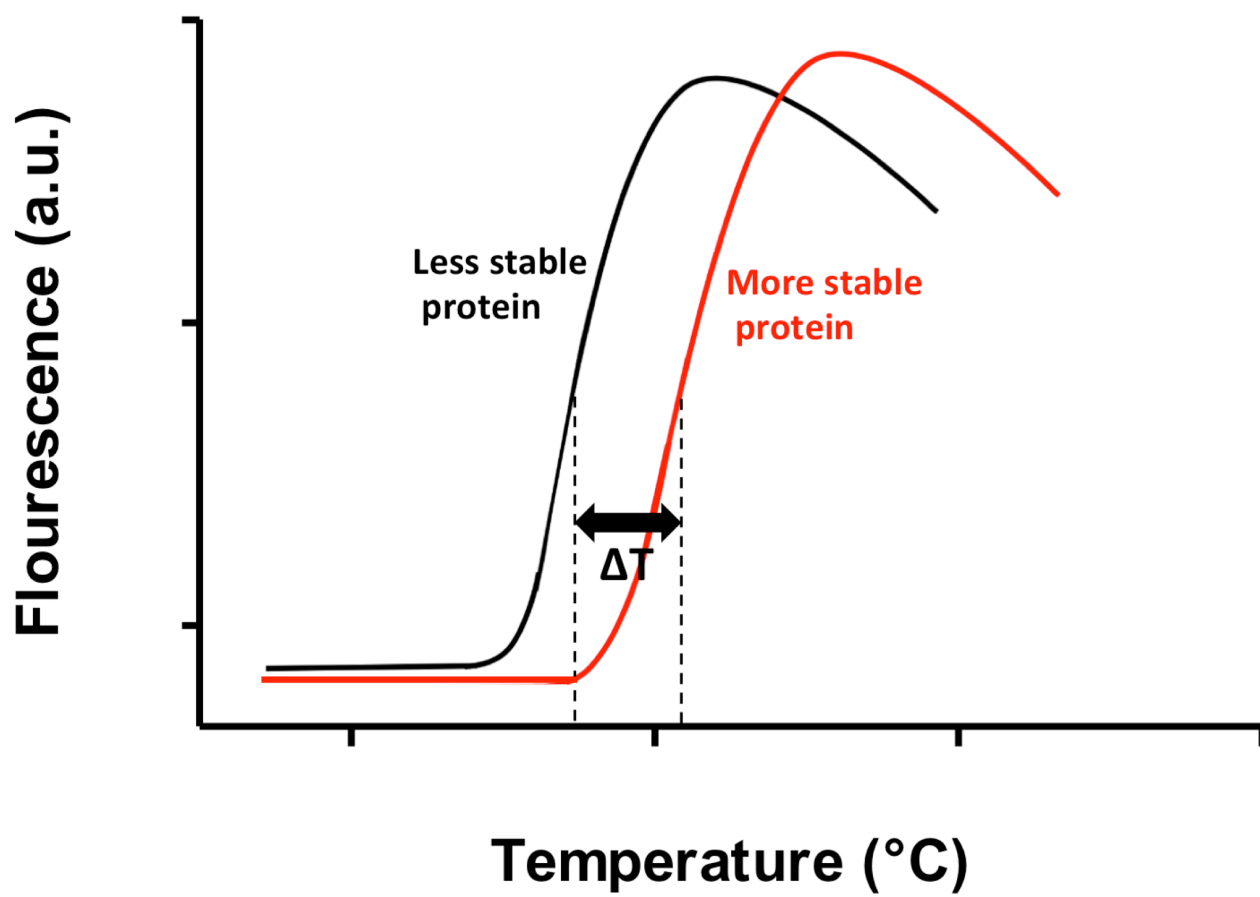

**(B)**

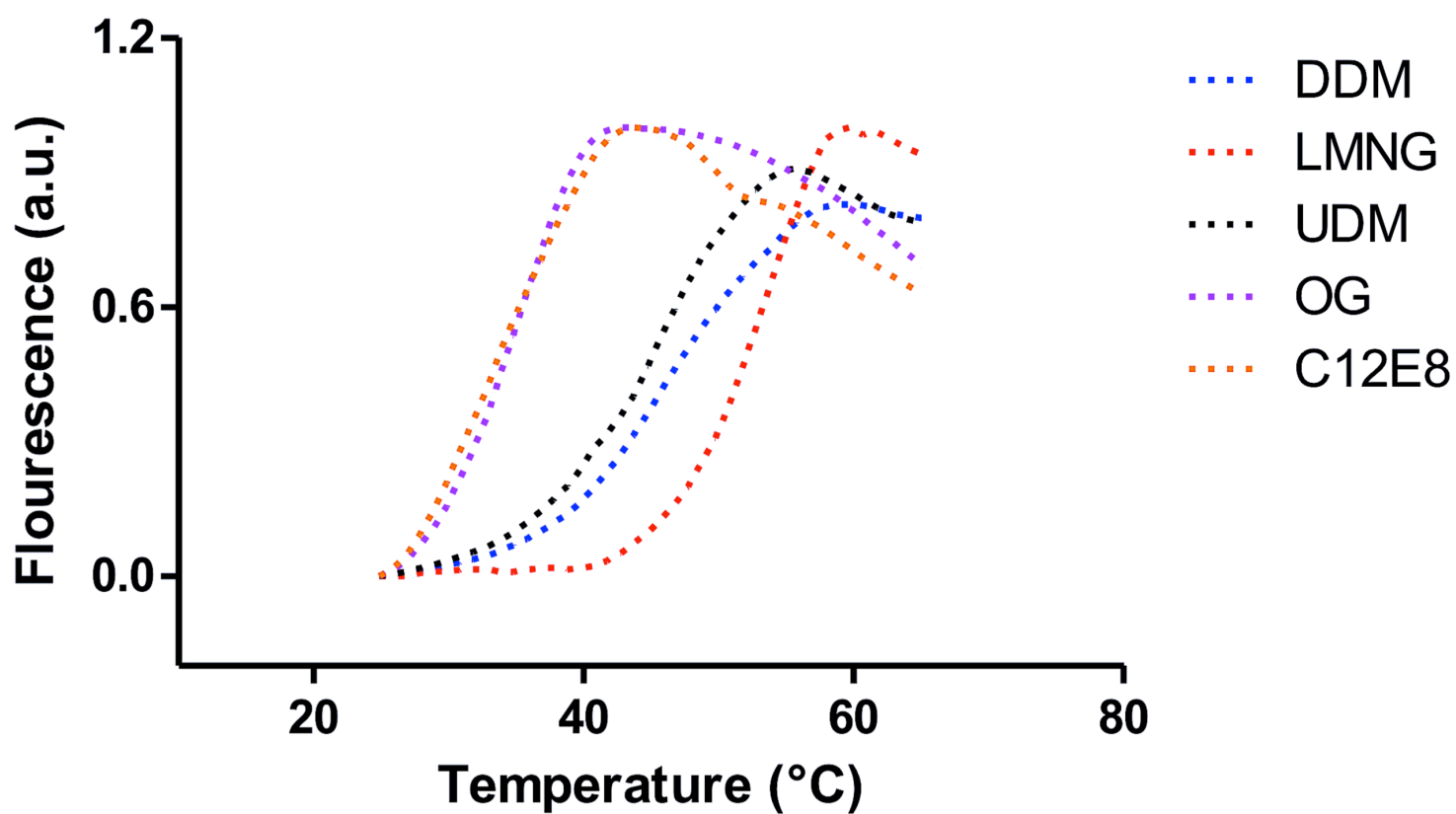

Supplement: Supplementary file 1 [file Image2.PDF]

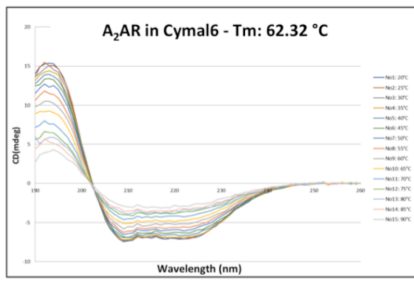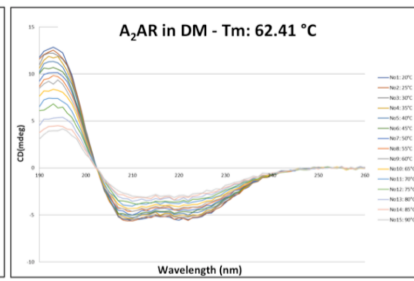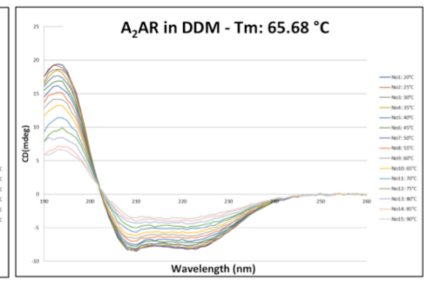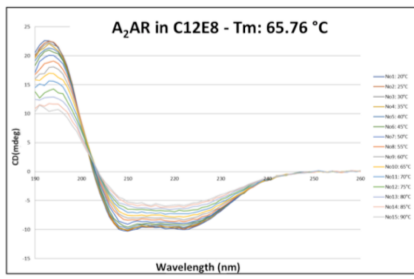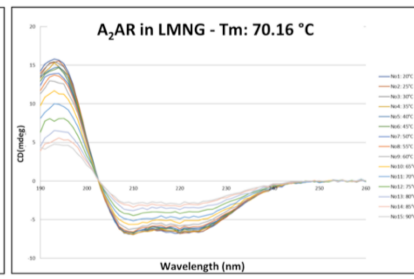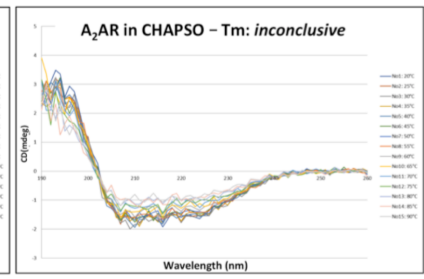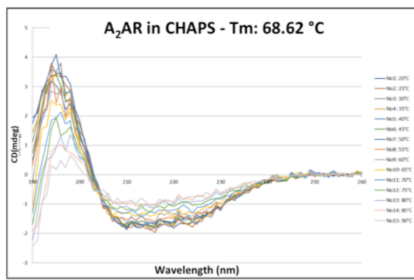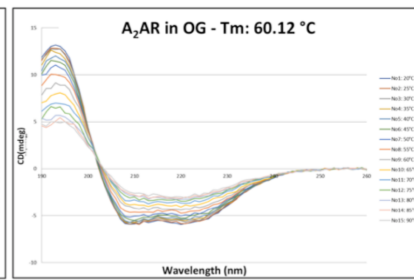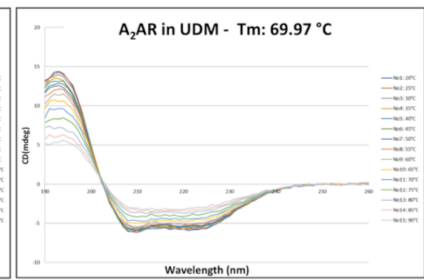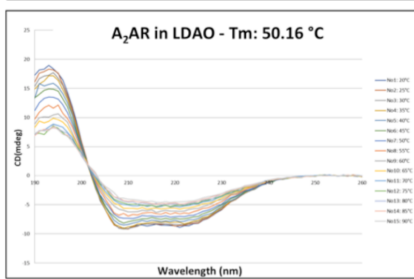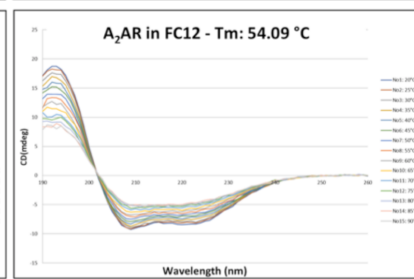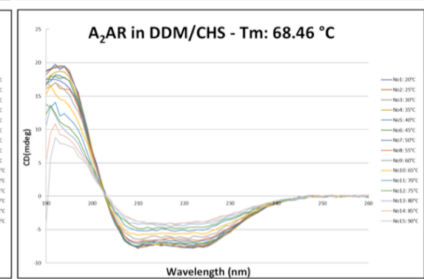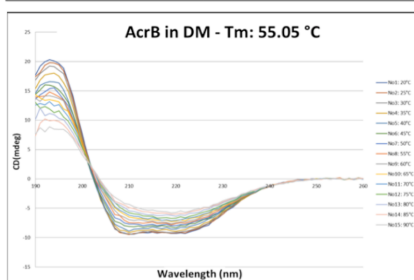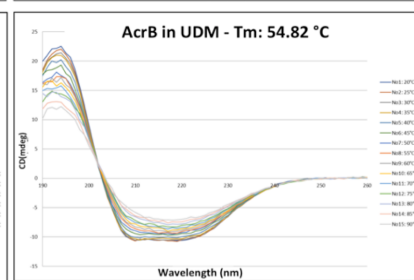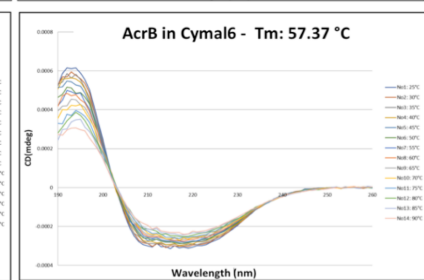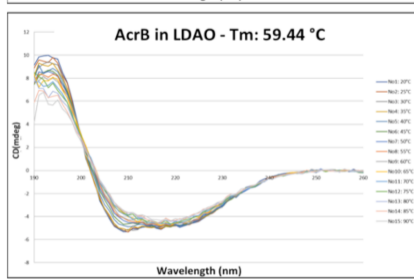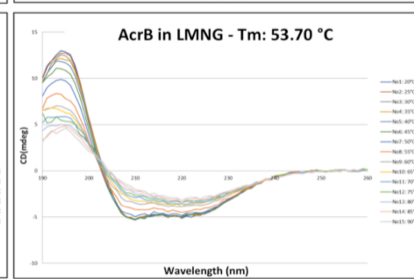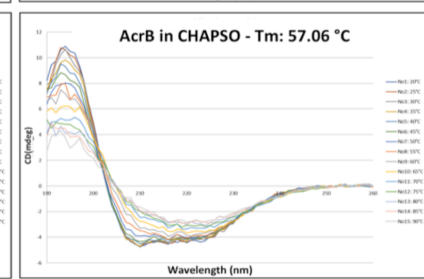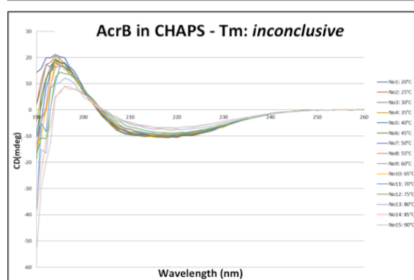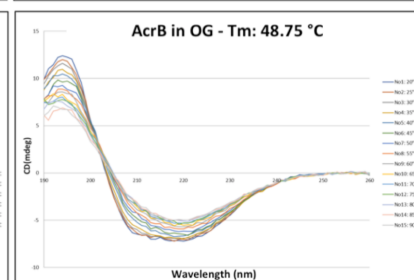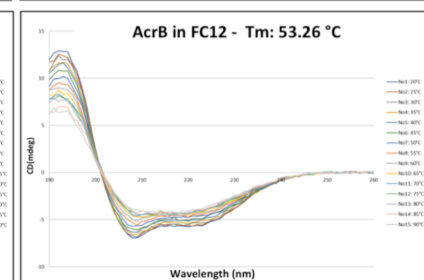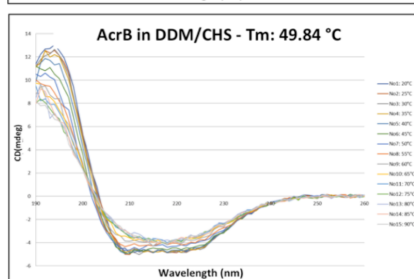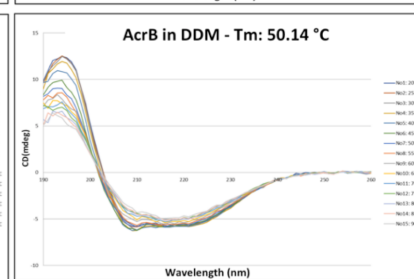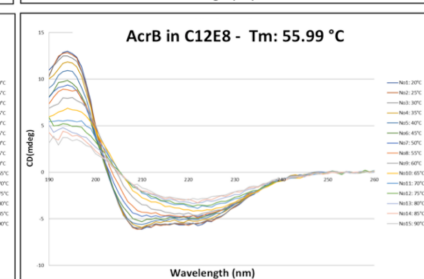

Supplement: Supplementary file 2 [file Image3.PDF]

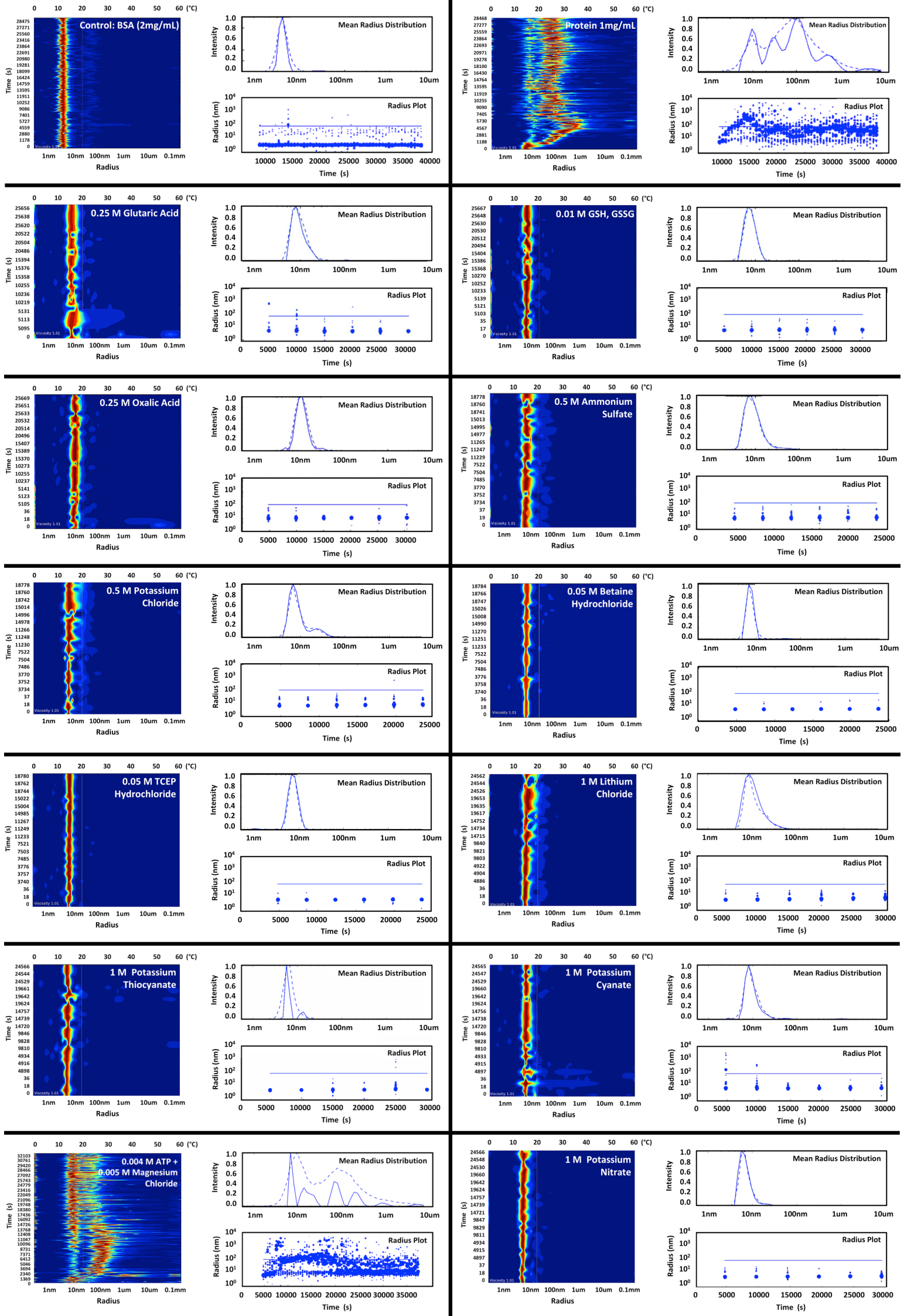

Supplement: Supplementary file 4 [file Image1.PDF]
