# Supplementary material for: Measuring Protein Aggregation and Stability Using High-Throughput Biophysical Approaches
Source: Front Mol Biosci. 2022 May 16;9:890862. doi: 10.3389/fmolb.2022.890862 (PMC9149252; doi:10.3389/fmolb.2022.890862)
Supplement: Supplementary file 3 [file DataSheet1.DOCX]

Supplementary Material

**Supplemental Methods**

**HTP *in situ* Dynamic Light Scattering (DLS) measurements:** For bovine serum albumin (BSA) (Thermo Fisher) control and protein-only sample, 1 μL drops were dispensed manually onto a 96-well microbatch hydrophobic plate (Douglas Instruments) covered in paraffin oil. The BSA was at 2 mg/mL and the protein in study, a bacterial ABC transporter, in 50 mM MES pH 6.5, 200 mM NaCl, 0.03% DDM at 1 mg/mL. Volumes of 0.2 μL of each sample (protein + screening conditions) were dispensed onto a 96-well microbatch hydrophobic plate (Douglas Instruments) using an Oryx8 robotic system (Douglas Instruments). The samples were covered with 10 µL of paraffin oil by the Oryx8 robotic system and the plate was then topped up with a further 2 mL of paraffin oil to provide a layer of oil with uniform height and surface. The *in situ* DLS experiments were carried out at 293 K using a SpectroLight 610 instrument (XtalConcepts GmbH, Hamburg, Germany) with 10 s scans and 6 repeated measurements per scan over a period of 9.6 h.

**CPM assay:** LacY (in 20 mM Tris pH 7.5, 150 mM NaCl, 0.03% DDM) was used for the experiment. CPM dye (4 mg/mL in DMSO) was first diluted 1/20 with buffer (20 mM Tris, pH 7.5, 150 mM NaCl) and 3x critical micelle concentration (CMC) of each detergent to be tested. The protein stock was also diluted with buffer and 3x CMC of each detergent to a final amount of 0.5 μg. The protein was incubated for 1 h before the addition of 7 μL of the thiol-specific fluorochrome N-[4-(7-diethylamino-4-methyl-3-coumarinyl)phenyl]maleimide (CPM) dye prepared with the respective detergent condition. The final volume for each well was 50 μL. After the addition of CPM dye, the plate was left to incubate for a further 15 min in the dark before running the thermal shift assay.

**HTP Synchrotron Radiation Circular Dichroism:** A_2_AR (in 40 mM Tris pH 7.4, 200 mM NaCl, 0.15% DM, 1 mM theophylline) and AcrB (in 10 mM Tris pH 7.5, 300 mM NaCl, 0.03% DDM, 5% (v/v) glycerol) were used at a concentration of approximately 0.7 mg/mL and 0.5 mg/mL, respectively. The detergents used were n-Dodecyl β-D-maltoside (DDM), DDM/Cholesteryl hemisuccinate (CHS), n-Decyl-β-D-Maltopyranoside (DM), n-Undecyl-β-D-Maltopyranoside (UDM), Lauryl Maltose Neopentyl Glycol (LMNG), n-Dodecyl-N,N-Dimethylamine-N-Oxide (LDAO), Cymal 6, Fos Choline 12, Octyl Glucose Neopentyl Glycol (OG), CHAPS, CHAPSO, Octaethylene Glycol Monododecyl Ether (C12E8) at 1% final concentration, with the exception of LMNG (0.25%), CHAPS (0.5%), CHAPSO (0.5%), and CHS (0.2%).The measurements were performed at B23 beamline (Module B end-station) at Diamond Light Source using a 6 cell Turret (rectangular cell pathlength: 0.02 cm, 0.05 cm, 1 cm).

**Supplementary Figure Captions**

**Supplementary Figure 1. Representative HTP *in situ* Dynamic Light Scattering (DLS) analysis of a bacterial ABC transporter in the presence of different buffer conditions**. The figure shows 14 results that include a control experiment using BSA (top-left panel), the protein in study in its purification buffer (top-right panel), and a selection of conditions from a 96-component screen. The inserts to the left of each panel show the time dependent size distribution plot in the form of a signal heat map (blue = low particle concentration, red = high particle concentration) and the inserts to the right show the *in situ* analysis graphs of the radius distribution and radial distribution plot (the blue spot diameter represents the relative scattered light intensity of the detected particles in arbitrary units).

**Supplementary Figure 2.** **Graphic representation of a typical melting curve obtained during a Differential Scanning Fluorimetry (DSF) experiment in the presence of a fluorescent dye**. (**A**) The black sigmoid curve represents a less stable protein with the red exemplifying a more stable protein. ΔT (change in transition midpoint for thermal unfolding) represents the variance between the melting temperatures (T_m_) at which each of the protein samples is 50% unfolded, also known as the thermal shift value. (**B**) Thermal stability profiles for the *Escherichia coli* lactose transporter (LacY) in the presence of different detergents using the CPM dye assay. Calculated T_m_ were determined by fitting the curves to a Boltzmann sigmoidal equation. The calculated values are as follows: 45.7 °C in the presence of n-Dodecyl β-D-maltoside (DDM); 50.9 °C in the presence of lauryl maltose neopentyl glycol (LMNG); 43.4 °C in the presence of n-Undecyl-β-D-Maltopyranoside (UDM); 32.2 °C in the presence of n-Octyl-β-D-Glucoside (OG); and 34.3 °C in the presence of dodecyl octaethylene glycol ether (C12E8).

**Supplementary Figure 3.** Synchrotron Radiation Circular Dichroism (SRCD) spectra of the human Adenosine 2A receptor (A_2_AR) and bacterial transporter AcrB in different detergents as a function of increasing temperature: 20 °C to 90 °C in 5 °C steps. In high-throughput protocols at least 96 conditions might be screened in each experimental run.
